# The p53 transcriptional response across tumor types reveals core and senescence-specific signatures modulated by long noncoding RNAs

**DOI:** 10.1073/pnas.2025539118

**Published:** 2021-07-29

**Authors:** Ephrath Tesfaye, Elena Martinez-Terroba, Jordan Bendor, Lauren Winkler, Christiane Olivero, Kevin Chen, David M. Feldser, Jesse R. Zamudio, Nadya Dimitrova

**Affiliations:** ^a^Department of Molecular, Cellular, and Developmental Biology, Yale University, New Haven, CT 06511;; ^b^Department of Cancer Biology, Abramson Family Cancer Research Institute, Perelman School of Medicine at the University of Pennsylvania, Philadelphia, PA 19104;; ^c^Department of Molecular, Cell, and Developmental Biology, University of California, Los Angeles, CA 90095;; ^d^Eli and Edythe Broad Center of Regenerative Medicine and Stem Cell Research, University of California, Los Angeles, CA 90095;; ^e^Jonsson Comprehensive Cancer Center, University of California, Los Angeles, CA 90095

**Keywords:** p53, tumor suppression, lncRNA, senescence, transcription

## Abstract

The p53 pathway is a universal tumor suppressor mechanism that limits tumor progression by triggering apoptosis or permanent cell cycle arrest, called senescence. In recent years, efforts to reactivate p53 function in cancer have proven to be a successful therapeutic strategy in murine models and have gained traction with the development of a range of small molecules targeting mutant p53. However, knowledge of the downstream mediators of p53 reactivation in different oncogenic contexts has been limited. Here, we utilized a panel of murine cancer cell lines from three distinct tumor types susceptible to alternative outcomes following p53 restoration to define unique and shared p53 transcriptional signatures. While we found that the majority of p53-bound sites and p53-responsive transcripts are tumor-type specific, analysis of shared targets identified a core signature of genes activated by p53 across all contexts. Furthermore, we identified repression of E2F and Myc target genes as a key feature of senescence. Characterization of p53-induced transcripts revealed core and senescence-specific long noncoding RNAs (lncRNAs) that are predominantly chromatin associated and whose production is coupled to *cis*-regulatory activities. Functional investigation of the contributions of p53-induced lncRNAs to p53-dependent outcomes highlighted *Pvt1b*, the p53-dependent isoform of *Pvt1*, as a mediator of p53-dependent senescence via *Myc* repression. Inhibition of *Pvt1b* led to decreased activation of senescence markers and increased levels of markers of proliferation. These findings shed light on the core and outcome-specific p53 restoration signatures across different oncogenic contexts and underscore the key role of the p53-*Pvt1b-Myc* regulatory axis in mediating proliferative arrest.

## Significance

The work by Tesfaye and colleagues defines universal and tumor type–specific features of the p53 tumor suppressor transcriptional network. This study determines a “core” signature of the p53 response across different oncogenic contexts, which defines a universal set of p53 target genes. In addition, this study clarifies the basis for outcome specificity downstream of p53 activation in different oncogenic contexts. We observe that while apoptosis in lymphoma cells is not primarily determined by p53’s transcriptional activity, p53 indirectly promotes senescence in lung adenocarcinoma and sarcoma cells by activating the *cis*-regulatory long noncoding RNA *Pvt1b*, which represses Myc levels and its proliferative function.

p53 is the most important tumor suppressor in the mammalian genome and the most frequently somatically mutated gene in human cancer (1). p53 operates as a stress-stabilized transcription factor that regulates a broad network of genes by binding to p53 motifs found in the promoters of target genes (2). Multiple studies have profiled the p53-regulated transcriptome across various biological contexts including the response to genotoxic stress (3–10) and oncogenic signaling (11–14). However, disentangling the contributions of cell-intrinsic versus stimuli-specific effects of p53 activation has proven challenging (15). While some studies have concluded that p53 function is primarily determined by the type of stressor used (13), others have suggested that cell identity plays a pivotal role in determining where p53 binds in the genome (16) and/or which genes are transactivated (3, 14).

Senescence and apoptosis are two alternative outcomes by which p53 limits aberrant growth. Apoptosis can lead to tumor regression through the permanent elimination of tumor cells (17, 18). Senescence can have pleiotropic effects on tumorigenesis by restricting proliferation (19) and signaling the immune clearance of tumor cells (20) or by triggering tumor-permissive immune suppression (21). The molecular cues determining why p53 initiates senescence downstream of p53 in certain contexts and apoptosis in others remain poorly understood.

Adding to the complexity of the p53 network, recent years have seen a growing appreciation for the roles of noncanonical players in the p53 network, including long noncoding RNAs (lncRNAs) (22). p53-activated lncRNAs have been reported to modulate gene expression by regulating p53 transcriptional activity (7, 23, 24), sequestering transcription factors (25), modulating p53-regulated enhancer activity (26, 27), or acting locally to fine-tune the expression of neighboring genes (28, 29). However, an integrated model for how lncRNAs contribute to the diverse tumor-suppressive functions of p53 is lacking.

To characterize the universal and context-specific signatures of the p53 response to oncogenic stress, we performed an integrated analysis of p53 binding sites and transcriptional outputs in response to oncogenic stress across a range of isogenic tumor types, cell lines, and p53-dependent outcomes. By investigating the contribution of lncRNAs to these signatures, we showed that the majority of p53-regulated lncRNAs exhibit *cis*-regulatory activities and highlighted *Pvt1b*, the p53-induced isoform of *Pvt1*, as a mediator of p53-dependent growth arrest.

## Results

### Genome-Wide Profiling of the p53 Response to Oncogenic Stress across Tumor Types.

**Fig. 1.**
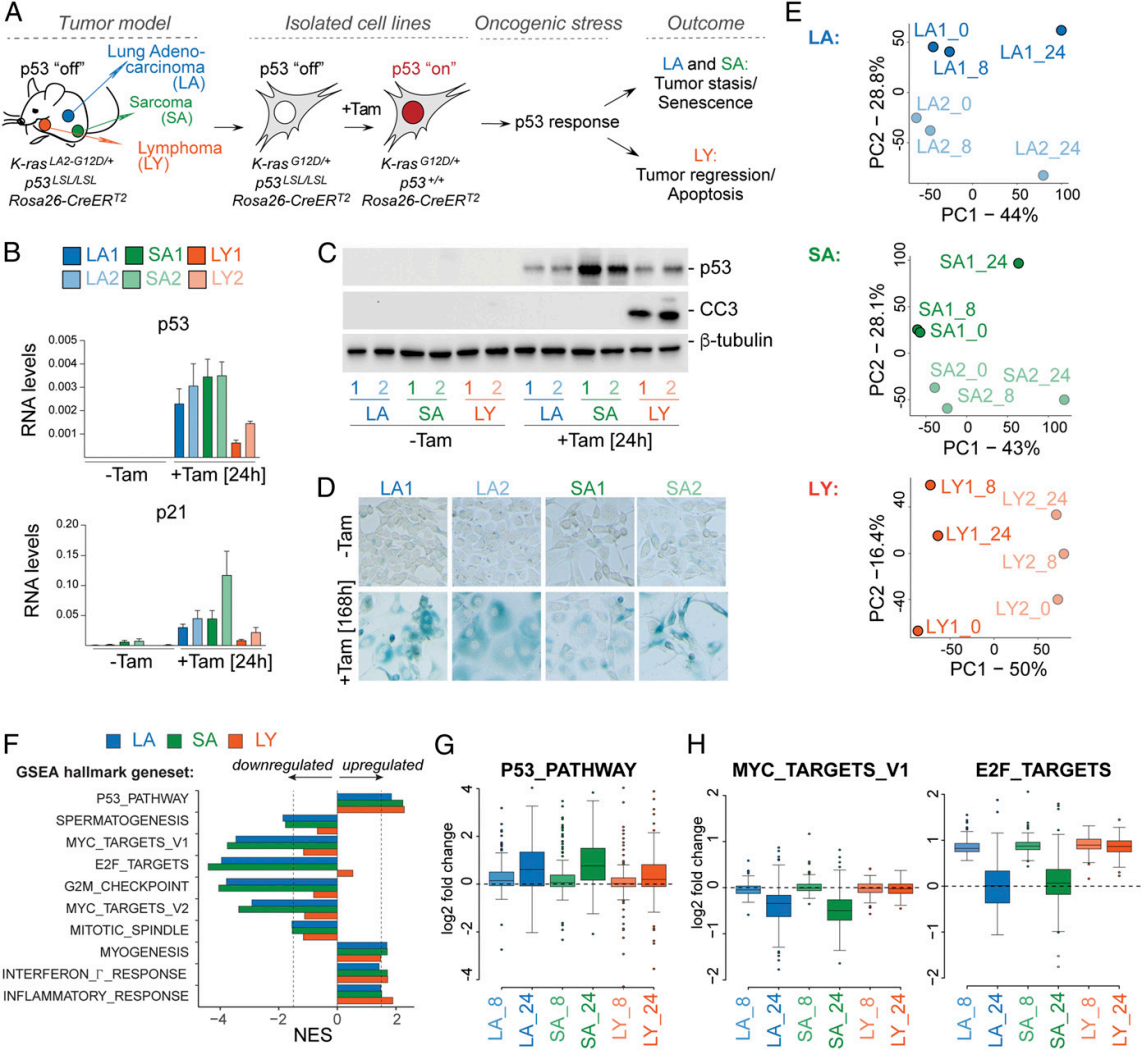
Characterization of the p53 transcriptional response to oncogenic stress across diverse tumor types and outcomes. (*A*) Diagram of the KPR murine model of cancer, tumor-derived cell lines, and tumor type–specific p53 outcomes upon Tam-mediated p53 restoration. (*B*) RT-qPCR analyses of *p53* and *p21* mRNA levels in six KPR cell lines following mock or Tam treatment. Data show mean ± SEM from *n* = 3 biological replicates. (*C*) Representative immunoblot analyses of p53 and cleaved caspase 3 (CC3) protein levels of cells in *B* from *n* = 3 biological replicates. β-tubulin is a loading control. (*D*) Representative images of senescence-associated β-galactosidase staining in indicated KPR cell lines and treatments from *n* = 3 biological replicates. (*E*) Principal component analysis (PCA) of RNA-seq from the indicated six KPR cell lines harvested at 0, 8, or 24 h after Tam treatment. (*F*) Normalized Enrichment Score (NES) of GSEA of the top 400 differentially expressed genes as determined by PCA in the three KPR tumor types. (*G* and *H*) p53-restoration–dependent expression changes of genes from indicated Hallmark GSEA gene sets in the indicated KPR tumor types and treatments relative to mock-treated samples.

To model the p53 response to oncogenic stress, we utilized a panel of cancer cell lines isolated from the *K-ras*^*LA2-G12D/+*^*; p53*^*LSL/LSL*^*; Rosa26-CreER*^*T2*^ (*KPR*) mouse model, which is an established tool for investigating the p53 tumor suppressor pathway across different tumor types (Fig. 1*A*) (18, 19). In this model, activation of the latent oncogenic *G12D* allele of K-ras occurs spontaneously in individual cells via somatic recombination and leads to the development of various tumor types including lung adenocarcinoma (LA), sarcoma (SA), and lymphoma (LY). Germ-line inactivation of the p53 tumor suppressor gene via transcriptional inhibition cassettes (*LoxP-STOP-LoxP* [*LSL*]) accelerates tumor formation and allows the propagation of cell lines isolated from these tumors in vitro (18). Previous studies have shown that endogenous p53 expression can be restored in tumor-bearing animals and in isolated cell lines by 4-hydroxytamoxifen (Tam)–activatable *CreER*^*T2*^ recombinase, which excises the *LSL* cassettes to allow *p53* transcription (18, 19). Following restoration, p53 is stabilized by the presence of oncogenic stress and promotes the expression of target genes, which results in distinct outcomes depending on tumor type (Fig. 1*A*). While LYs undergo apoptosis in vitro and tumor regression in vivo (18), LAs and SAs senesce in vitro and exhibit tumor stasis in vivo (19). In this study, we focused on a panel of six cell lines isolated from *KPR* tumors, including two LA-derived (LA1 and LA2), two SA-derived (SA1 and SA2), and two LY-derived (LY1 and LY2) cell lines. Restoration of p53 expression and activation of the p53 pathway was confirmed across all Tam-treated cell lines by immunoblotting for p53 and RT-qPCR for p53 and its target gene, *Cdkn1a/p21* (Fig. 1 *B* and *C*). As expected from previous studies (18, 19), we observed induction of apoptosis by the detection of cleaved caspase 3 (CC3) in LY, but not LA or SA cell lines, at 24 h after p53 restoration (Fig. 1*C*). Activation of senescence in LA and SA cell lines was detected by staining for β-galactosidase activity at 168 h following p53 restoration (Fig. 1*D*).

### Universal and Senescence-Specific p53 Signatures in Response to Oncogenic Stress.

To characterize the p53 response to oncogenic stress across these three different tumor types and two distinct cellular outcomes, we performed RNA sequencing (RNA-seq) of untreated and Tam-treated cells harvested at early (8 h) or late (24 h) time points following p53 restoration. Principal component analysis (PCA) revealed distinct global gene expression signatures in the different tumor types, with cell lines from the same tumor type clustering closely regardless of treatment (*SI Appendix*, Fig. S1). Examination of the heterogeneity within each tumor type indicated a robust p53 transcriptional signature, with variations evident between untreated samples and samples harvested at 24 h following p53 restoration (LA: PC1 44%; SA: PC1 43%; and LY: PC2 16.4%), allowing us to study the p53-dependent response to oncogenic stress across these different contexts (Fig. 1*E*).

Clustering of the top 400 genes from PCA (*SI Appendix*, Fig. S2 *A*–*C*), gene set enrichment analysis (GSEA) (Fig. 1*F*), and transcription factor (TF) target analysis (*SI Appendix*, Fig. S2*D* and Dataset S1) of genes differentially expressed at 24 h after Tam largely revealed two main categories of genes. On the one hand, gene sets related to p53 function were significantly enriched across all cell lines, consistent with a shared p53-driven transcriptional signature (Fig. 1*F* and *SI Appendix*, Fig. S2*D*). Importantly, genes from the HALLMARK_P53_PATHWAY gene set displayed a significant and measurable early induction at 8 h following p53 restoration, which was, on average, ∼12% of the transcript levels measured at the 24-h time point (Fig. 1*G*). On the other hand, we identified sets of down-regulated genes, which were enriched for factors that positively regulate cell cycle progression (E2F targets, Myc targets, G2/M checkpoint factors, and mitotic spindle checkpoint factors) (Fig. 1*H* and *SI Appendix*, Fig. S2 *A*–*D*). In contrast to the p53 target genes, Myc and E2F target genes were unresponsive at the early time point of Tam treatment and were found to be preferentially down-regulated at 24 h following p53 restoration (Fig. 1*H*). These findings established the presence of an early and universal p53-dependent transcriptional activation program in response to oncogenic signaling, which is largely independent of cell line– and tumor type–specific variations. In addition, these data suggested inputs from additional transcriptional networks, including Myc and E2F, in a subset of the cell types.

Across these analyses, we noted that LA and SA shared similar expression patterns, while LY exhibited distinct gene expression signatures (Fig. 1 *F* and *H* and *SI Appendix*, Fig. S2 *A*–*D* and Dataset S2). Namely, we found that at 24 h following p53 restoration, LA and SA cell lines consistently clustered together and showed comparable enrichments for related GSEA and TF target gene sets. In particular, Myc and E2F target genes showed significant overlap between LA and SA cell lines (MYC_TARGETS, V1, *P* < 2e-16; E2F_TARGETS, *P* < 1e-16) but were not enriched in LY. The similarity of the p53 response between these tumor types was further confirmed by correlation analysis, which revealed that LA and SA expression profiles were positively correlated to a much higher extent than any LY-related correlations (Pearson correlation: LA–SA, *r* = 0.67, *P* = 0.2; LA–LY, *r* = 0.59, *P* = 0.05; SA–LY, *r* = 0.44, *P* = 0.05) (*SI Appendix*, Fig. S2*E*). We examined whether this was due to a shared senescence response that LA and SA cell lines activate following p53 restoration. Indeed, analyses of curated GSEA gene sets revealed that LA and SA cell lines shared a notable senescence signature (TANG_SENESCENCE_UP, TANG_SENESCENCE_DOWN), which was absent from LY-derived cells (*SI Appendix*, Fig. S2*F*). We concluded that there is a senescence-specific transcriptional signature during the p53 response to oncogenic stress, which features repression of Myc- and E2F-controlled cell cycle genes. Importantly, the senescence signature was only evident at 24 but not at 8 h following p53 restoration and was not present in cells that undergo apoptosis, differentiating it from the direct p53-dependent transcriptional activation response (*SI Appendix*, Fig. S2*F*).

### Identification of Core and Senescence-Specific p53 Targets.

**Fig. 2.**
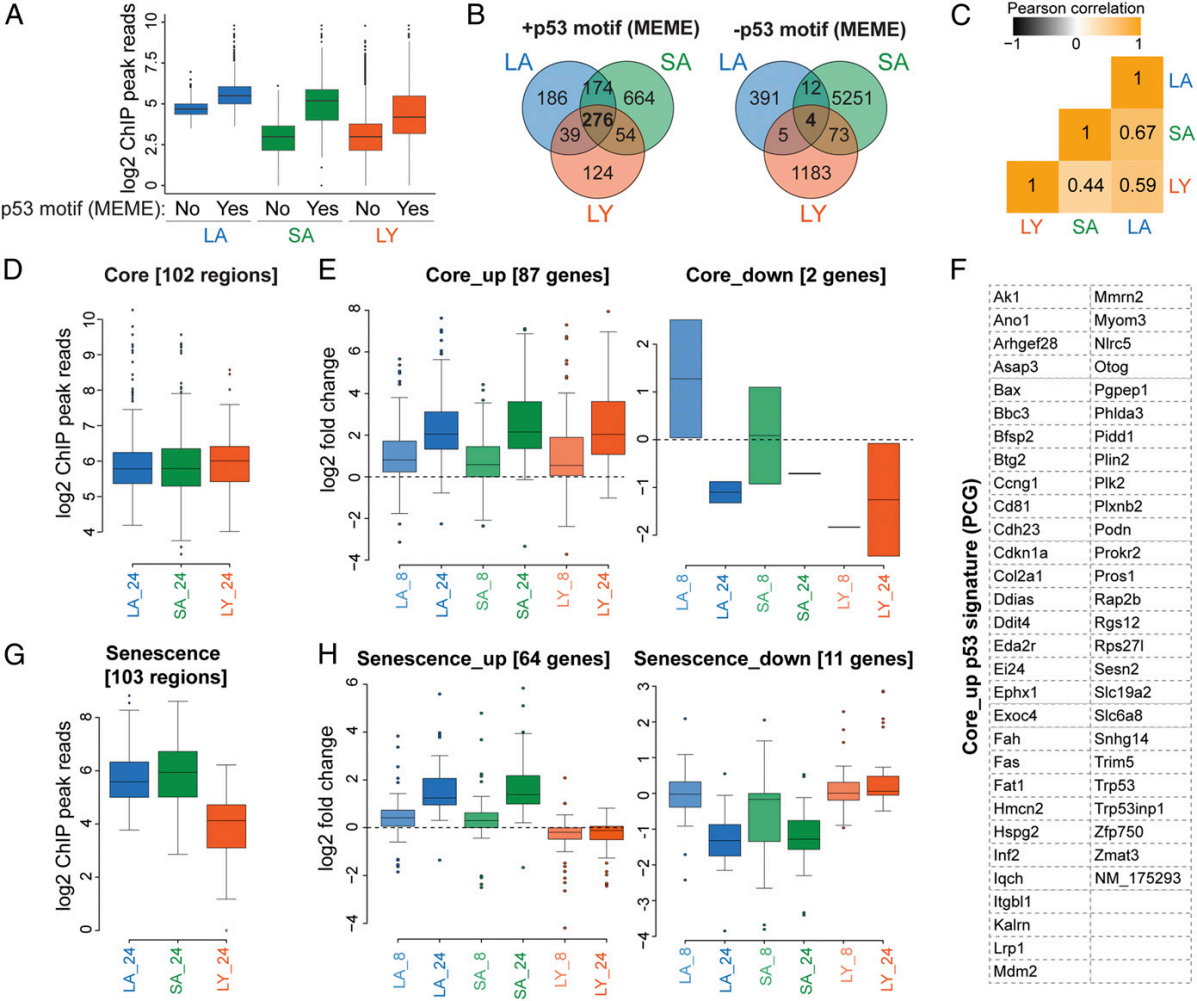
Identification of p53-regulated core and senescence-specific targets. (*A*) Normalized p53 ChIP-seq read densities in indicated KPR tumor types. Peaks are categorized by absence (No) or presence (Yes) of the p53 motif, determined by MEME analysis. (*B*) Venn diagrams of p53 peak distributions across KPR tumor types, grouped by peaks with (*Left)* or without *(Right)* apparent p53 motifs, determined by MEME analysis. (*C*) Pearson correlation matrix of indicated p53 ChIP-seq profiles; numbers show *r*-values. (*D*) Normalized p53 ChIP-seq reads at 102 core genomic regions. (*E*) p53-restoration–dependent fold change in the expression levels of 89 core genes corresponding to the regions in *D* in indicated KPR tumor types and time points of Tam treatment. (*F*) Table of 56 core PCGs induced following p53 restoration across all KPR tumor types. (*G*) Normalized p53 ChIP-seq reads at 103 senescence-specific regions. (*H*) p53-restoration–dependent fold change in the expression levels of 75 senescence-specific genes corresponding to the regions in *G* in indicated KPR tumor types and time points of Tam treatment.

To determine whether p53 contributes directly to these universal and outcome-specific transcriptional responses, we mapped p53 binding sites by chromatin-immunoprecipitation and sequencing (ChIP-seq) in the tumor cell lines at 24 h following mock treatment or Tam-mediated p53 restoration (Dataset S3). Consistent with high-confidence identification of p53 binding sites, the top multiple expectation maximizations for motif elicitation (MEME) motif in each sample was the p53 motif. Although peak numbers varied between cell types, increased number of peaks in ChIP-seq experiments did not result in the identification of more bound sites with p53 motifs (*SI Appendix*, Fig. S3*A*), which could be related to ChIP efficiency or suggest varying numbers of weaker and potentially nonfunctional p53 genomic interactions. As expected, based on normalized read numbers within peaks, p53 peaks with p53 motifs displayed nearly twofold higher read density compared to those without a motif, suggesting stronger binding (Fig. 2*A* and Dataset S4). Furthermore, by examining shared p53-bound regions between cell types, we observed a higher overlap in peaks with p53 motifs compared to peaks without apparent motifs by MEME analysis (Fig. 2*B* and Dataset S5). From these analyses, we identified 276 motif-containing genomic regions enriched over background across all cell types, indicating the presence of a core p53-bound signature (Fig. 2*B*). We also noted 174 p53-bound regions shared between LA and SA but not LY samples and a strong correlation between the ChIP-seq profiles of LA and SA cell lines, suggesting direct p53 transcriptional input into the senescence outcome (Fig. 2 *B* and *C*).

We next determined how these binding events related to transcriptional output by integrating the p53 binding and transcriptome profiles (*SI Appendix*, Fig. S3*B*). Using stringent criteria to define regions based on significant p53 binding by ChIP-seq, presence of p53 motifs, and detection of transcriptional output by RNA-seq, we identified 102 core and 103 senescence-specific regions bound and activated by p53 (Dataset S6). Core regions showed nearly uniform levels of p53 binding across all three tumor types and gave rise to a comparable transcriptional output at early and late time points of p53 restoration (Fig. 2 *D* and *E*). Importantly, this analysis led us to define a high-confidence set of 56 protein-coding genes (PCG), which represent the core p53 response to oncogenic stress independent of cell line and tumor type variability (Fig. 2*F*).

In addition, we observed senescence-specific regions, which showed similar levels of p53 binding in LA and SA but a 3.3-fold reduction in read density in LY (Fig. 2*G*). p53 binding was accompanied by a pronounced up-regulation of the corresponding 64 genes at 8 (*P* < 1.6 × 10^−5^) and 24 (*P* < 1.6 × 10^−6^) hours following p53 restoration in both LA and SA but not in LY cell lines (Fig. 2*H*). Similarly, we observed a small set of genes, which were preferentially responsive in LY at 8 and 24 h following p53 restoration (*SI Appendix*, Fig. S3*C*). These data revealed the presence of an early and direct p53-dependent transcriptional response that might set the stage for the activation of prosenescence and proapoptotic pathways (Dataset S6).

### Core and Senescence-Specific lncRNAs in the p53 Oncogenic Stress Response.

We noticed that 31 out of 87 core genes (36%) and 19 out of 64 senescence-specific genes (30%) were lncRNAs, suggesting broad contributions from this class of noncoding transcripts to the p53 response (Dataset S7). Two of the core lncRNAs (*lincRNA-p21* and *Pvt1*) have previously been characterized as p53 targets (29–31), while the remaining were novel transcripts that have not been associated with the p53 pathway. Novel lncRNAs were named antisense (as) according to the gene they overlapped in the antisense orientation, upstream antisense (ua) to indicate divergent transcripts, or long intergenic noncoding RNAs (lincRNA) according to their nearest neighbor.

**Fig. 3.**
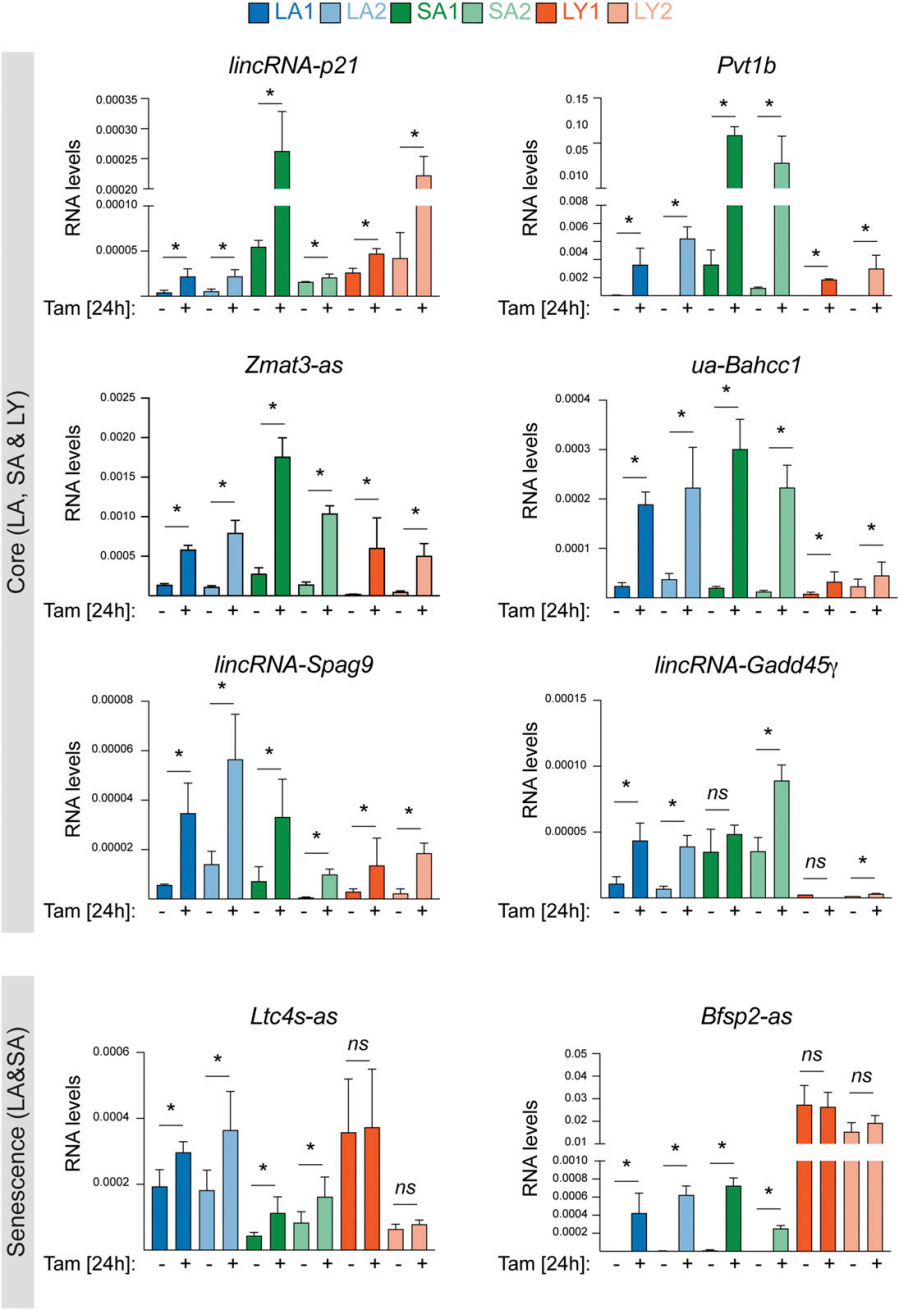
Oncogenic stress drives the p53-dependent induction of core and senescence-specific lncRNAs. Validation by RT-qPCR of indicated core and senescence-specific lncRNAs in indicated mock and Tam-treated KPR cells. Data show mean ± SEM from *n* = 3 biological replicates; *P < 0.05, *ns* = not significant, paired *t* test.

Validation by RT-qPCR confirmed induction of *lincRNA-p21* and the p53-dependent isoform of *Pvt1*, Pvt1b (29), across the full panel of cell lines (Fig. 3). Similarly, we observed p53-dependent up-regulation for *Zmat3-as*, *ua-Bahcc1*, *lincRNA-Spag9*, and *lincRNA-Gadd45*γ across all tumor types, whereas *Ltc4s-as* and *Bfsp2-as* showed p53-dependent activation in the senescence-prone LA and SA cell lines (Fig. 3). These observations suggested that lncRNAs might contribute to both the core- and outcome-specific p53 responses.

### Widespread *Cis*-Regulatory Activities of lncRNAs in the p53 Pathway.

**Fig. 4.**
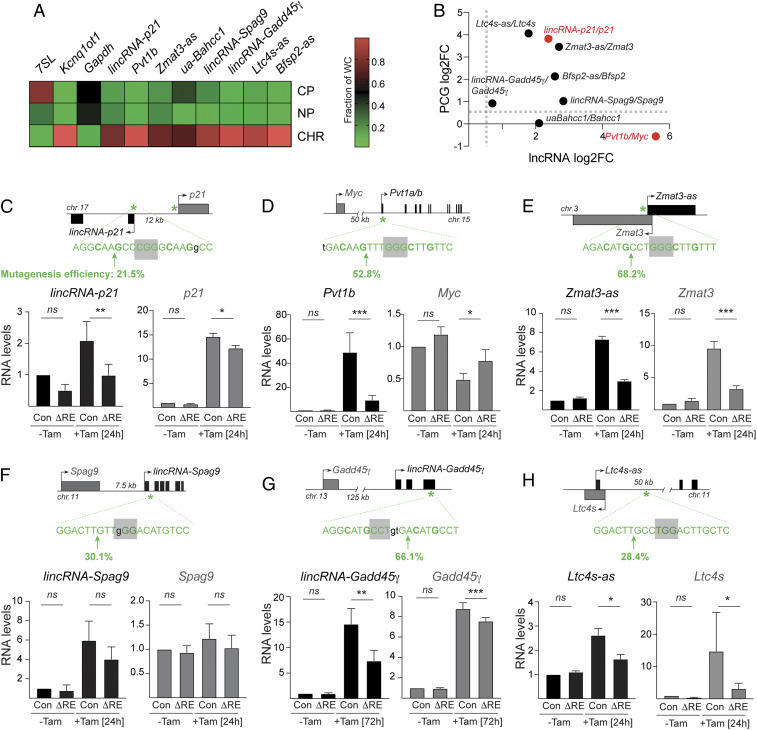
p53-induced lncRNAs are chromatin enriched and linked to the expression of their neighboring genes. (*A*) Heatmap of subcellular enrichment of indicated lncRNAs in LA1 cells treated with 24-h Tam, determined by RT-qPCR and normalized to total cellular RNA in *n* = 2 biological replicates. *Rn7sl, Kcnq1ot1*, and *Gapdh* are fractionation controls. CP = cytoplasmic. NP = nucleoplasmic. CHR = chromatin. (*B*) Correlation plot of the fold changes of indicated lncRNAs and corresponding neighboring PCG mRNAs in LA1 cells treated with 24-h Tam. Data show mean values from *n* = 3 biological replicates. (*C*–*H*, *Top*) Schematic of lncRNA–PCG genomic loci, p53RE location and sequence, and CRISPR-Cas9 mutagenesis strategy of lncRNA-associated p53REs highlighting protospacer adjacent motif (PAM) (gray box) and predicted Cas9 cleavage site (green arrow). Mutagenesis efficiency indicates the fraction of cells with indels in the p53RE, determined by TIDE analysis in *SI Appendix*, Fig. S3. (*Bottom*) RT-qPCR analysis of relative RNA levels of indicated lncRNAs and corresponding PCG mRNAs in indicated cell lines and treatments. Data show mean ± SEM from *n* = 3 to 5 biological replicates; *P < 0.05, **P < 0.01, ***P < 0.001, *ns* = not significant, paired *t* test.

Subcellular fractionation in LA cells revealed that all of the validated p53-induced lncRNAs were enriched in the chromatin fraction, as previously shown for *lincRNA-p21* and *Pvt1b* (Fig. 4*A*) (28, 29). Interestingly, the neighboring PCGs of seven of the validated p53-induced lncRNAs also appeared to be p53-responsive (Fig. 4*B*). This correlation in p53-dependent expression was positive for the majority of lncRNA–PCG pairs, with the exception of *Pvt1b*, which showed negative correlation with its neighbor, *Myc*, as previously described (29).

lncRNAs have been reported to play diverse roles in transcriptional networks, but one emerging theme is that lncRNAs can act as local regulators of gene expression (32). To further examine the relationship between lncRNAs and their neighboring PCGs, we designed a CRISPR/Cas9 mutagenesis strategy to specifically inactivate lncRNAs by targeting lncRNA-associated p53 response elements (p53REs), which are required for p53-dependent expression. We successfully designed specific guide RNAs (gRNAs) to target the p53REs of six lncRNAs (Fig. 4 *C*–*H*, *Top*) and obtained 20 to 70% mutagenesis efficiency in LA1.1 cells, determined by TIDE (Tracking Indels by Decomposition) analysis (*SI Appendix*, Fig. S4 *A*–*F*). Mutagenesis of the p53REs led to a significant decrease in the expression levels of five out of the six lncRNAs (ΔRE, Fig. 4 *C*–*H*, *Bottom*). These included the previously characterized *lincRNA-p21* and *Pvt1b*, as positive controls, as well as the novel *Zmat3-as*, *lincRNA-Gadd45*γ, and *Ltc4s-as.* As expected from previous work showing *cis* regulation, we found that down-regulation of *lincRNA-p21* in Tam-treated ΔRE-*lincRNA-p21* cells correlated with reduced *p21* levels compared to Tomato (Tom)-targeting control (Fig. 4*C*) (28), whereas down-regulation of *Pvt1b* in Tam-treated ΔRE-*Pvt1b* cells led to increased *Myc* expression (Fig. 4*D*) (29). Next, we found that mutagenesis of the p53RE associated with *Zmat3-as* in ΔRE-*Zmat3-as* cells, *lincRNA-Gadd45γ* in ΔRE-*lincRNA-Gadd45*γ cells, and *Ltc4s-as* in ΔRE-*Ltc4s-as* cells also resulted in reduced expression of their neighboring genes, *Zmat3*, *Gadd45*γ, and *Ltc4s-as,* respectively, compared to control cells (Fig. 4 *E*, *G*, and *H*). These data confirmed that these lncRNAs are direct transcriptional targets of p53 and that they are coregulated with their neighbors in a p53-dependent manner.

Given the role of *lincRNA-p21* in promoting the expression of its neighboring gene, *p21,* and the role of *Pvt1b* in suppressing the transcription of its neighbor, *Myc*, we considered the possibility that *Zmat3-as*, *lincRNA-Gadd45*γ, and *Ltc4s-as* might also play functional roles in modulating the expression of their neighboring genes. While *Zmat3-as* and *Ltc4s-as* overlap their neighboring PCGs, rendering functional dissociation of the lncRNA and overlapping PCG challenging, *lincRNA-Gadd45*γ and *Gadd45*γ are 125 kilobases apart. To distinguish whether the p53 binding site or lncRNA production contributed to the regulation of *Gadd45*γ, we performed CRISPR-based epigenetic modulation of the *lincRNA-Gadd45*γ transcript. First, we inhibited *lincRNA-Gadd45*γ expression via transcriptional interference by targeting Cas9 downstream of the transcription start site (TSS) (*SI Appendix*, Fig. S5*A*, *Top*). We observed a 50% decrease in *lincRNA-Gadd45*γ levels and a concomitant 30% decrease in *Gadd45γ* expression in Tam-treated cells (*SI Appendix*, Fig. S5*A*). In the converse experiment, we used the CRISPR-synergistic activation mediators (SAM) system (33) to induce expression of *lincRNA-Gadd45*γ 28-fold in the absence of p53 expression (*SI Appendix*, Fig. S5*B*, *Top*). This led to a corresponding ninefold induction of *Gadd45*γ (*SI Appendix*, Fig. S5*B*). We concluded that transcription of *lincRNA-Gadd45*γ was both necessary and sufficient to modulate *Gadd45*γ expression, analogous to *lincRNA-p21* and *Pvt1b*.

### Functional Contributions of *Cis*-Regulatory lncRNAs to the p53 Response.

**Fig. 5.**
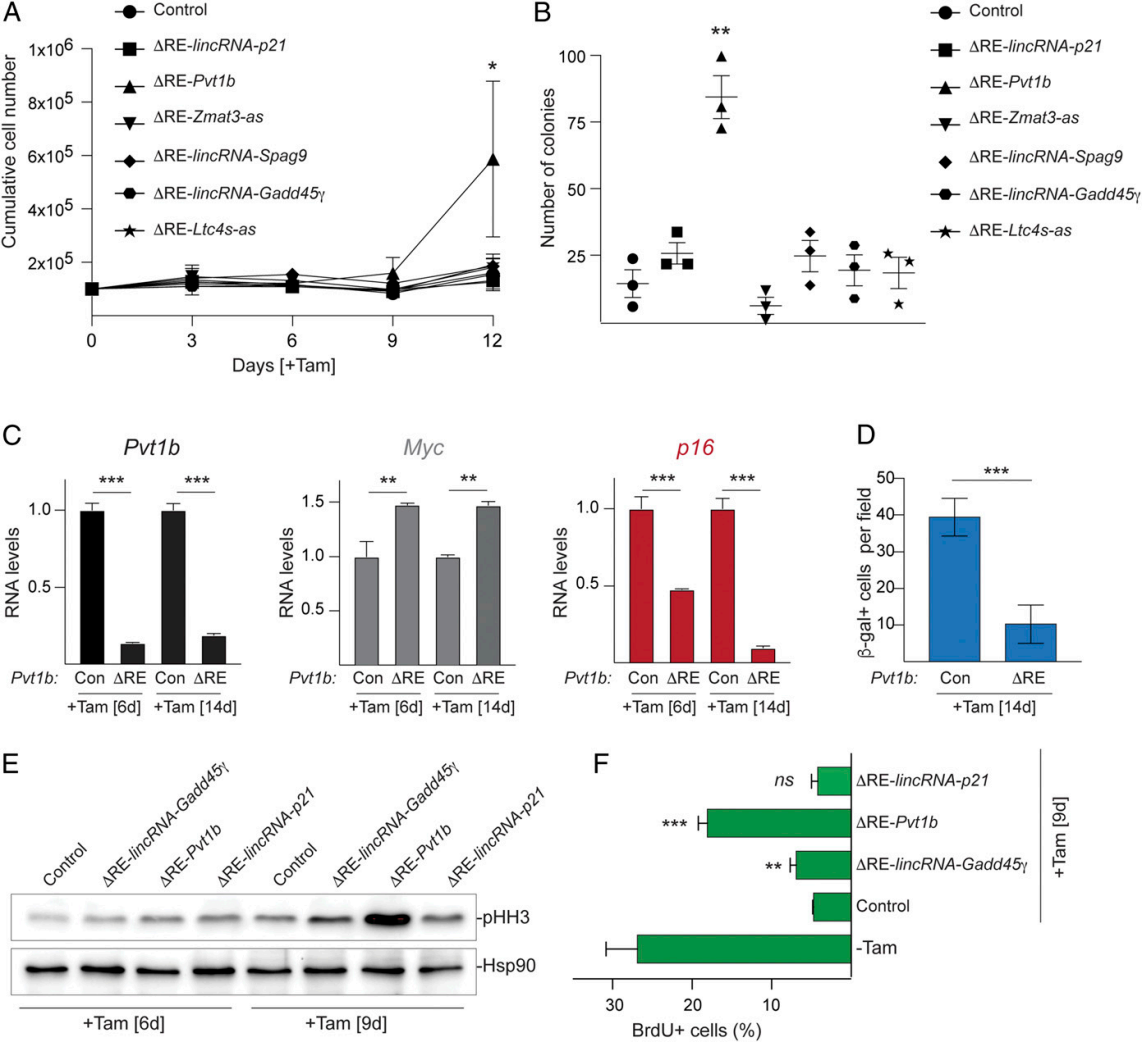
*Cis*-repression of *Myc* by *Pvt1b* contributes to the senescence p53 outcome in response to oncogenic stress. (*A*) Cumulative cell numbers over time of indicated Tam-treated LA1.1 cell lines. Data represent mean ± SEM (*n* = 3 biological replicates). (*B*) Quantification of colony formation in indicated Tam-treated LA1.1 cell lines. Data represent mean ± SEM from *n* = 3 biological replicates. (*C*) RT-qPCR analysis of relative RNA levels of *Pvt1b*, *Myc*, and *p16* in indicated LA1.1 cell lines and treatments. Data show mean ± SEM from *n* = 3 biological replicates. (*D*) Bar graph of the number of β-galactosidase–positive cells (β -gal+) per field of indicated LA1.1 cells analyzed at 14 d after Tam treatment. Data represent mean ± SEM from *n* = 3 biological replicates. (*E*) Representative immunoblot analyses of pHH3 in indicated LA1.1 cell lines and treatments from *n* = 3 biological replicates. Hsp90 is a loading control. (*F*) Bar graph of the fraction of BrdU-positive (BrdU+) cells in fluorescence-activated cell sorting (FACS) analysis of indicated LA1.1 cell lines, harvested untreated or 9 d after Tam treatment. Data represent mean ± SEM from *n* = 3 to 4 biological replicates, *P < 0.05, **P < 0.01, ***P < 0.001, *ns* = not significant, paired *t* test between Tam-treated ΔRE and control cells.

Finally, we examined whether core and senescence-specific lncRNAs contributed to the senescence outcome of p53 restoration. We performed growth curve and colony formation assays with control and ΔRE-mutant LA1.1 cells. As expected, control cells expressing Tom-targeting single guide RNA (sgRNA) underwent permanent cell cycle arrest following Tam-mediated p53 restoration and failed to grow or form colonies (Fig. 5 *A* and *B*). Consistent with previous findings, we found that ΔRE-*Pvt1b* cells partially overcame the permanent growth arrest and formed colonies (Fig. 5 *A* and *B*) (29). In contrast, inhibition of *lincRNA-p21*, *Zmat3-as*, *lincRNA-Gadd45γ,* and *Ltc4s-as* was not sufficient to overcome senescence-induced arrest (Fig. 5 *A* and *B*). The lack of measurable phenotypic effect for most of the tested lncRNAs in these assays is likely due to functional redundancy within the p53 network. On the other hand, the effect of *Pvt1b* inhibition on partially overcoming senescence reinforces a previously proposed model in which *Pvt1b* plays a mediating role at the intersection of the p53 and Myc transcriptional networks by repressing *Myc* transcription and thus limiting proliferative capacity (29).

To further investigate the effects of *Pvt1b* inhibition on the activation of senescence, we compared ΔRE and control LA1.1 cells over 2 wk following Tam-mediated p53 restoration (Fig. 5 *A* and *B*). We observed that the reduction of *Pvt1b* and the increase in *Myc* RNA levels persisted over time, suggesting a long-term role for *Pvt1b* in suppressing *Myc* expression (Fig. 5*C*). In addition, we found a significant reduction in the senescence markers, p16 and β-galactosidase activity (Fig. 5 *C* and *D*), and a marked increase in the proliferation markers, phosphorylated histone H3 (pHH3) and bromodeoxyuridine (BrdU) incorporation (Fig. 5 *E* and *F*), in ΔRE-*Pvt1b* cells compared to controls. These data confirm a role for *Pvt1b* in mediating p53-activated senescence in response to oncogenic stress.

## Discussion

By investigating the p53-regulated transcriptome in six independent cell lines derived from lung adenocarcinomas, sarcomas, and lymphomas, we generated a rich dataset that revealed the significant extent of cell line– and tumor type–dependent heterogeneity in the p53 transcriptional response to oncogenic stress. Next, by focusing on shared targets across these independent contexts, we identified 56 protein-coding genes and 31 lncRNAs as universal targets of p53 transcriptional activity. Among these genes, we found known mediators of checkpoint function and apoptosis downstream of p53, including Cdkn1a/p21, Mdm2, Bax, Pidd1, and others (15). The list also contains factors involved in processes such as autophagy (Ei24, Sesn2), metabolism (Pgpep1, Fah), tumor microenvironment (Col2a1, Hspg2, Itgbl1), and immune response (Cd81), which represent emerging features of the p53 response to oncogenic stress (34). We propose that this “core” p53 signature defines the key functions of p53 in the context of oncogenic stress and will be instrumental to efforts to assess the efficacy of therapeutic reactivation of p53 in human cancer.

The diverse outcomes of p53 activation in our model system also allowed us to address whether activation of p53 target genes determines apoptosis- or senescence-specific transcriptional programs in different tumor types. By comparing the p53 binding patterns and p53-dependent gene expression changes in lung adenocarcinoma and sarcoma cell lines (which undergo senescence) to lymphoma cell lines (which undergo apoptosis), we found limited evidence that p53-dependent early gene activation plays a significant role in specifying these outcomes. This conclusion is consistent with an emerging model in which context-specific p53 cellular responses arise from cell- or stressor-specific factors such as mitochondrial apoptotic priming (35). On the other hand, it is conceivable that p53-dependent long-term transcriptional changes may drive phenotypic changes, such as those associated with senescence, which our present analysis would have missed. It is also possible that weaker p53 sites or sites without p53 motifs may contribute to the heterogeneity of the p53 response across biological contexts. The extent of occupancy of such sites may further depend on p53 protein levels, which can vary extensively between different tumor types, as observed in our panel of cell lines.

Interestingly, we found that down-regulation of E2F and Myc target genes is a key feature of the p53 transcriptional response. These findings are consistent with the recently reported interplay between the p53 and Myc pathways (29, 36) and with the proposed role for the DREAM/E2F complex in mediating gene repression downstream of p53 (37). Our observation that repression of E2F and Myc target genes is a late event following p53 restoration supports the previous conclusions that these effects are likely indirect and do not reflect direct repressive activities by p53. Importantly, we show that down-regulation of E2F and Myc target genes is a feature specific to senescence, suggesting that suppression of genes involved in cellular proliferation plays an important role in promoting this outcome downstream of p53.

Our work also led to the identification of multiple p53-regulated lncRNAs and offered insights into the roles of these lncRNAs in the context of the p53 pathway. We observed that lncRNAs constitute a significant portion of the core (36%) and senescence-specific (30%) signatures, raising a question of whether p53-regulated lncRNAs may have broad functional contributions to the p53 response. Curiously, a number of previously reported p53-induced lncRNAs, such as *Neat1* (38), *PANDAR* (25), *LED* (26), and *DINO* (39), were not found in our dataset, likely due to species-, cell line– or stimuli-dependent variability in the p53 pathway. We found that many of the lncRNAs, identified in this study, including *lincRNA-Gadd45γ,* show evidence for *cis*-regulatory activities. These data are consistent with our previously published work on *lincRNA-p21* (28) and *Pvt1b* (29) and expand our understanding of the widespread *cis* activities of lncRNAs in the p53 pathway and, possibly, other transcriptional networks. It is important to note, however, that the genetic and epigenetic approaches used to modulate lncRNA expression in this study do not distinguish between whether the lncRNAs themselves or the act of their transcription are necessary and sufficient for *cis*-regulation (32).

Our findings suggest that the production of p53-regulated lncRNAs acts primarily to fine-tune local gene expression but has limited contributions to the biological outcomes of p53 reactivation. Indeed, with the exception of *Pvt1b*, we found that lncRNA inhibition does not affect the growth arrest outcome of p53 activation in lung adenocarcinoma cells. We believe that the functional significance of *Pvt1b* in this context is mediated through its *cis*-inhibitory effect on *Myc* (29), which in turn promotes the senescence outcome.

It is important to note that the *cis*-inhibitory function of the lncRNA *Pvt1b* appears to be unique in the p53 network and not a general mechanism by which p53 enacts gene repression, as we did not find additional examples of p53-induced *cis*-regulatory repressive lncRNAs. We speculate that inhibitory relationships, such as those between *Pvt1b* and *Myc*, require the involvement of additional molecular machinery to reverse the activating input of p53 to a locally repressive output. We imagine that such relationships are likely to have evolved at key regulatory nodes of transcriptional pathways and are, therefore, predicted to enact important downstream functions.

## Materials and Methods

### Cell Lines and Treatments.

KPR cell lines were previously established from LA1 and LA2, SA1 and SA2, or LY1 and LY2 tumors (19). LA and SA cells were maintained in Dulbecco′s Modified Eagle′s Medium supplemented with 10% fetal bovine serum (FBS), 50 U/mL penicillin/streptomycin (pen/strep), 2 mM L-glutamine, 0.1 mM nonessential amino acids, and 0.055 mM β-mercaptoethanol. LY cells were cultured in Iscove's Modified Dulbecco's Medium (GIBCO) supplemented with 10% FBS, 50 U/mL pen/strep, and 0.055 mM β-mercaptoethanol. All cells were grown at 37 °C in a humidified incubator with 5% CO_2_. Puromycin-sensitive LA1.1 cells were generated as previously described (29). To excise the LSL cassette, cells were treated with 0.5 µM Tam (Cayman Chemical Company).

Mutagenesis was performed by infecting LA1.1 cells with gRNAs cloned in BRD001 lentiviral vector (gift from the Broad Institute, Massachusetts Institute of Technology). For CRISPR interference experiments, 15-mer “dead RNA” (dRNA) expressed from BRD0001 were targeted to a region downstream of the TSS. CRISPR activation was achieved by infecting previously generated LA1-Cas9-GFP cells with the Lenti-SAM-Hygro vector coexpressing dRNA-MS2 and MBP-p65-Hsp1 (29). All control and targeting gRNA and dRNA sequences are listed in *SI Appendix*, Table S8.

Lentivirus was produced in 293 cells (ATCC) by cotransfecting the lentiviral constructs with Δ8.2 (Addgene No. 8455) and VSV-G (Addgene No. 8454) viral packaging constructs. Virus-containing supernatants supplemented with 4 μg/mL polybrene (Millipore Sigma) were used to infect cells in two to three consecutive lentiviral infections delivered at 24-h intervals. Following infections, cells were selected with 5 μg/mL puromycin (Sigma-Aldrich) or 0.8 μg/mL hygromycin (Roche).

### DNA Analysis.

To isolate genomic DNA, LA1.1 cells expressing control or p53RE-targeting gRNAs were harvested 10 d after infection and resuspended in Genomic DNA Lysis Buffer (1 M Tris⋅HCl, pH 8.0; 0.5 M EDTA, pH 8.0; 20% sodium dodecyl sulfate, 5 M NaCl) with 1 mg/mL Proteinase K (Roche) and incubated at 55 °C overnight. DNA was neutralized with 3 M sodium acetate by inversion, precipitated with isopropanol, and resuspended in Tris-EDTA, pH 8.0. To determine mutagenesis efficiency, a region spanning each p53RE was amplified by conventional PCR (PrimeStar HS mix, Takara Bio), with primer sequences listed in Dataset S8. PCR products were cleaned (QIAquick PCR Purification Kit) and analyzed by Sanger sequencing with sequencing primers listed in Dataset S8. The type and frequency of mutations were analyzed using the TIDE web tool (http://tide.nki.nl) comparing wild-type and mutant cells (40).

### RNA Analysis.

At 8 or 24 h after mock or Tam treatment, total RNA was isolated from the panel of six KPR cell lines using TRIzol reagent (Invitrogen). RT-qPCR analysis was performed with primers listed in Dataset S8. RNA expression levels were determined relative to the housekeeping gene Gapdh. For RNA-seq, RNA was treated with DNaseI and subjected to RNeasy purification (Qiagen). Ribosomal RNA was removed by RiboMinus Eukaryotic Kit for RNA-Seq (Invitrogen), and complementary DNA library preparation was performed using TruSeq Stranded mRNA Library Prep (Illumina) and submitted for high-throughput sequencing.

### Immunoblotting.

Antibodies used for immunoblotting were as follows: p53 (1C12, Cell Signaling Technology), cleaved caspase 3 (Asp175, Cell Signaling Technology), β-tubulin (9F3, Cell Signaling Technology), pHH3 (Ser10, Cell Signaling Technology), and HSP90 (C45G5, Cell Signaling Technology). Immunoblots were visualized using Amersham ECL Prime Western Blotting Detection Reagent (GE Healthcare).

### ChIP-seq.

At 24 h after mock or Tam treatment, LA2, SA1, and LY1 cells were cross-linked in 1% methanol-free formaldehyde (Thermo Fisher Scientific). ChIP was performed as previously described (28) with p53 antibody (P53-CM5P-L, Leica). Input and p53 IP samples were barcoded and submitted for high-throughput sequencing.

### Subcellular Fractionation.

Subcellular fractionation was performed as previously described in LA1 cells (41). Subcellular RNA enrichment patterns were determined by RT-qPCR with primers, listed in Dataset S8. Fraction threshold cycle (Ct) values were normalized to whole-cell Ct values. Rn7s1, Gapdh, and Kcnq1ot1 served as fractionation quality controls.

### Cellular Proliferation, Colony Formation, and Senescence Assay.

Cellular proliferation was assessed by plating 1 × 10^5^ LA1.1 cells in 6-cm plates in the presence of Tam. The media was replaced every 3 d with fresh media. Cells were harvested and counted at indicated times, and cumulative cell numbers were plotted over time. For colony formation assays, 4 × 10^5^ control or p53RE mutant LA1.1 cells were plated in 6-cm plates in the presence of Tam. At 14 d after plating, colonies were stained in 0.5% crystal violet and 25% methanol for 10 min, followed by extensive washes in ddH_2_O prior to visualization. The senescence assay was performed at pH 5.5, as previously described (42), with cells grown in regular media or media supplemented with 0.5 to 1 μM Tam for the indicated time. To compare the frequency of senescent cells, cells were fixed at 14 d after Tam treatment, and the number of β-galactosidase–positive cells per field was scored in at least five fields from *n* = 3 biological replicates.

### BrdU Assay.

BrdU was added to the cells at a final concentration of 10 µM and incubated for 60 min. Cells were washed and fixed in cold ethanol for 30 min, followed by an incubation with 2N HCl/Triton ×100 for 30 min at room temperature to denature the DNA and neutralization with 0.1 M Na_2_B_4_O_7_. Next, cell pellets were incubated with 20 µL anti-BrdU-fluorescein isothiocyanate (Becton Dickinson) at 4 °C overnight. Finally, cells were resuspended in phosphate-buffered saline containing 5 µg/mL propidium iodide (Thermo Fisher Scientific) and analyzed by flow cytometry (BD FACS LSR Fortessa ×20).

### Bioinfomatic Analyses.

RNA-seq reads were mapped to mm10 with TopHat (43) to Gencode transcript annotation (M9), and transcripts were annotated with StringTie (44). Gene transfer format (GTF) files were generated by merging StringTie GTFs. Messenger RNA (mRNA)-seq reads were counted that overlapped exons or intronic parts using custom scripts and bedtools multicov using the -split option. To compare mRNA levels in different conditions, normalization factors were determined for each sample using a DESeq2-like normalization approach (45). PCA was done in R. Gene sets from the Molecular Signature Database were downloaded from the GSEA (46) webpage (https://www.gsea-msigdb.org/gsea) (47). Significantly changing gene sets were identified using false discovery rate (FDR) < 0.05 in all three or in any pairs of samples at 24 h after p53 restoration.

ChIP-seq reads were mapped to the mm10 genome assembly with Bowtie 1, allowing up to two mismatches (48). Reads with a multiplicity greater than 1 were randomly assigned to a single genomic region. Peaks of p53 binding were identified using MACS2 (49) using pooled negative control samples for the respective tumor type. The presence of p53 motif was determined using FIMO in MEME suite. Peaks with FDR < 0.01 and a log2-enrichment ≥5-fold compared to pooled negative control were considered for further analysis. Positions of peaks that were significant in any of the tumor cell lines were merged. Next, reads overlapping these peak positions were counted for each cell line and treatment. The read count of each peak in each condition was normalized to the sum of reads overlapping those peaks that were among the top 30% of peaks in every condition ranked by the number of reads overlapping a peak. ChIP-seq peaks were assigned to the closest gene and termed intragenic, if the region overlapped or was located within 2 kb upstream of an annotated transcript, or intergenic, if they were not within 2 kb.

## Supplementary Material

Supplementary File

Supplementary File

Supplementary File

Supplementary File

Supplementary File

Supplementary File

Supplementary File

Supplementary File

Supplementary File

## Data Availability

RNA-seq and ChIP-seq data generated in this study can be accessed at the National Center for Biotechnology Information Gene Expression Omnibus under accession no. GSE163404 (50).
