# Supplementary material for: The p53 transcriptional response across tumor types reveals core and senescence-specific signatures modulated by long noncoding RNAs
Source: Proc Natl Acad Sci U S A. 2021 Jul 29;118(31):e2025539118. doi: 10.1073/pnas.2025539118 (PMC8346867; doi:10.1073/pnas.2025539118)
Supplement: Supplementary File [file pnas.2025539118.sapp.pdf]

SI Appendix

Legends to SI Datasets

SI Dataset 1. Differential expression of genes across cell lines and timepoints (Log2FC [+Tam/-Tam]) determined by RNA-seq. Log2 fold change of differentially expressed genes in LA1 (KPR8), LA2 (KPR10), SA1 (306), SA2 (928), LY1 (3472), and LY2 (3940) KPR cell lines treated with 8 or 24 hours Tam relative to mock treated samples.

SI Dataset 2. RNA classification of differential genes based on 2-fold change in a biological replicate. Differentially expressed genes categorized by activation (up; log2FC>1) or repression (down; log2FC<1) at 24 hours Tam treatment relative to mock treated samples and KPR tumor type grouping (p53_all (LA, SA, LY) and sen (senescence; LA and SA)).

SI Dataset 3. p53 bound regions with p53 motif and calls in each tumor type indicated. Genomic coordinates (mm10) and FIMO motif analysis for p53 bound regions from ChIP-seq analysis of LA (KPR10), SA (306), and LY (3472) KPR cell lines treated with 24h Tam.

SI Dataset 4. p53 bound genes with motif (genic and intergenic). Genes associated with all p53 motif-containing p53 bound regions. Peaks were categorized as genic if overlapping or within 2 kb upstream of an annotated transcript. All other peaks were considered intergenic. For intergenic peaks, the nearest gene is indicated.

SI Dataset 5. Universal and senescence bound regions and genes, determined by ChIP-seq. Genomic coordinates and peak-associated genes for p53 motif-containing p53 bound regions, grouped by KPR tumor type ChIP profiles: Universal (U; LA, SA, LY), senescence-preferential (S; LA and SA).

SI Dataset 6. RNA and p53 binding-based calls of genes in the universal and senescence response. p53 bound regions and associated genes differentially expressed upon 24h Tam treatment, grouped by core (LA, SA, LY) or senescence-specific (LA and SA) expression patterns.

SI Dataset 7. LncRNAs in universal and senescence response. Transcript IDs and assigned names for p53-induced lncRNAs identified in this study, grouped by expression patterns. Novel transcripts were assigned names in reference to nearest protein coding gene and lncRNA biotype. Bolded lncRNAs were validated in this work.

SI Dataset 8. Oligonucleotide, sgRNA, and dRNA sequences used in this study.

Tesfaye et al., Supplemental Figure 1

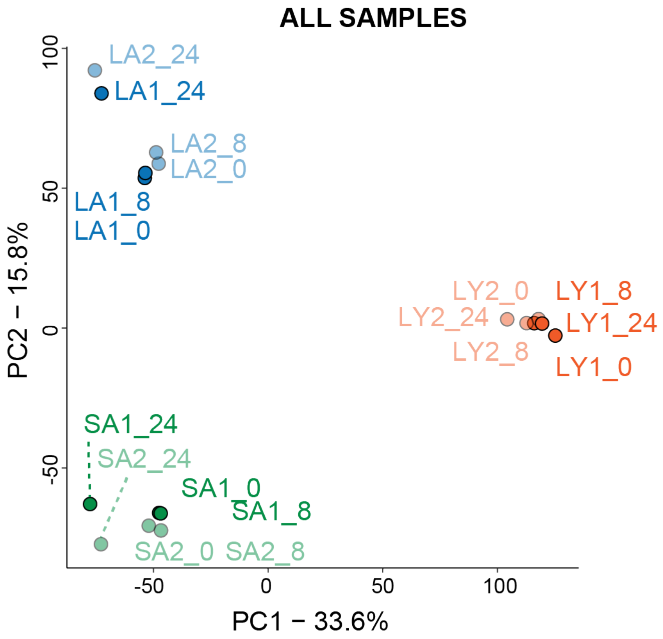

Figure S1. Principal component analysis (PCA) of RNA-seq from all six KPR cell lines harvested at 0, 8, or 24 h post Tam treatment.

Tesfaye et al., Supplemental Figure 2

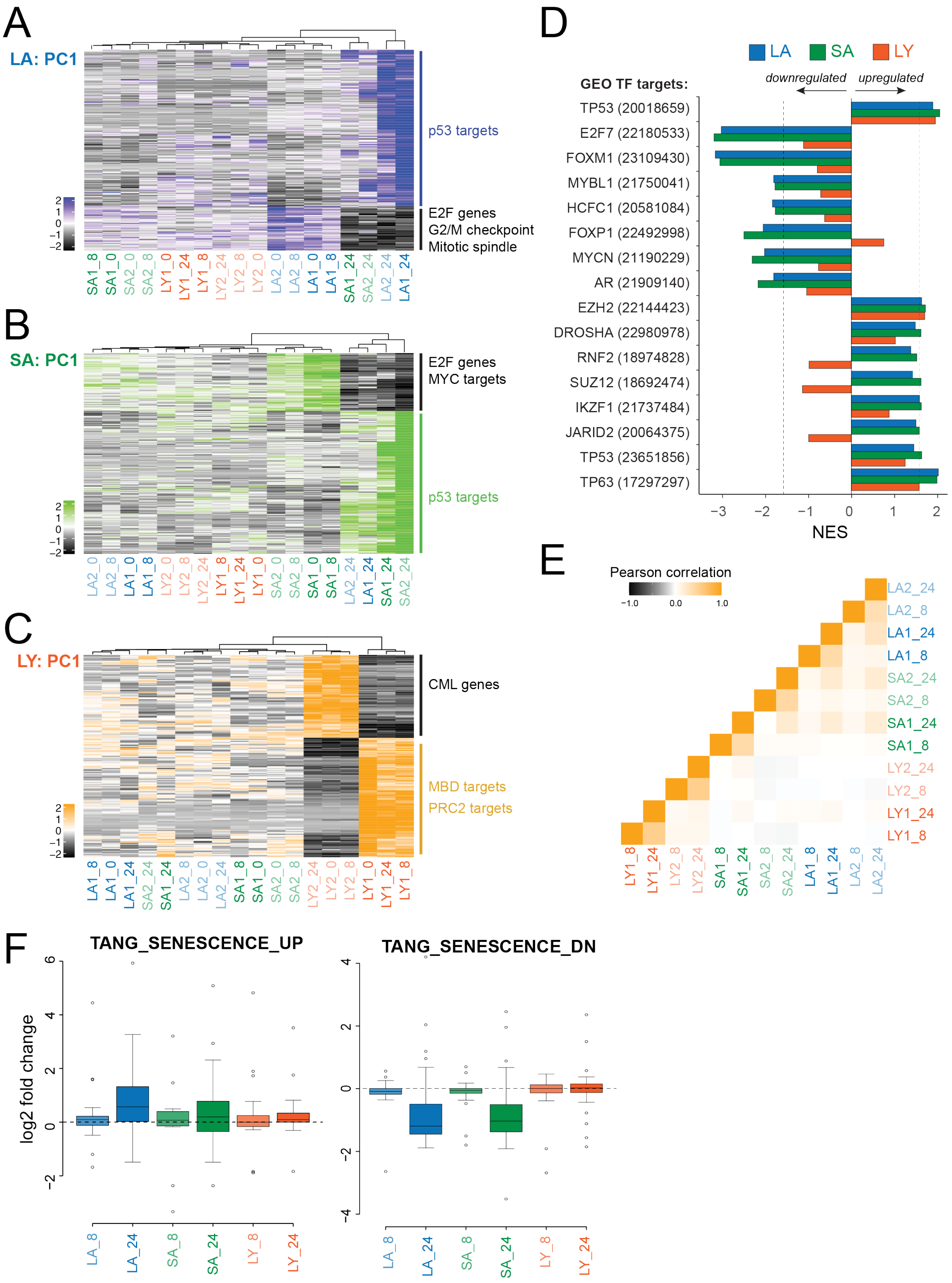

Figure S2. Additional characterization of the p53-responsive transcriptome across KPR tumor types and cell lines. *(A-C)* Clustering of top 400 differentially expressed genes across indicated KPR cell lines and treatments. Ranked gene lists were determined by LA (A), SA (B), and LY (C) intra-tumor type PCA. *(D)* Gene Expression Omnibus (GEO) Transcription Factor (TF) target analysis of the top 400 differentially expressed genes in indicated KPR tumor types. GEO accession numbers for each TF dataset are noted. (*E*) Pearson correlation matrix of gene expression profiles of indicated KPR cell lines and treatments. (*F*) p53-restoration-dependent expression changes of genes from indicated Molecular Signature Database GSEA gene sets in the indicated KPR tumor types and treatments relative to mock treated samples.

Tesfaye et al., Supplemental Figure 3

A

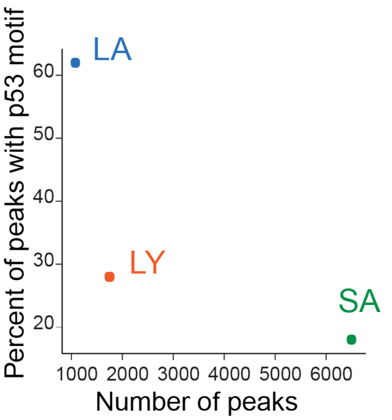

B

p53 RNA signature		With p53 motif: Genic	With p53 motif: Intergenic	Without p53 motif: Genic+intergenic	Total
Core (LA&SA&LY)	Up	87	11	957	1055
	Down	2	2	360	364
Senescence (LA&SA)	Up	64	10	2134	2208
	Down	18	11	1236	1265
LY only	Up	24	6	433	463
	Down	7	5	391	403

p53 ChIP signature

C

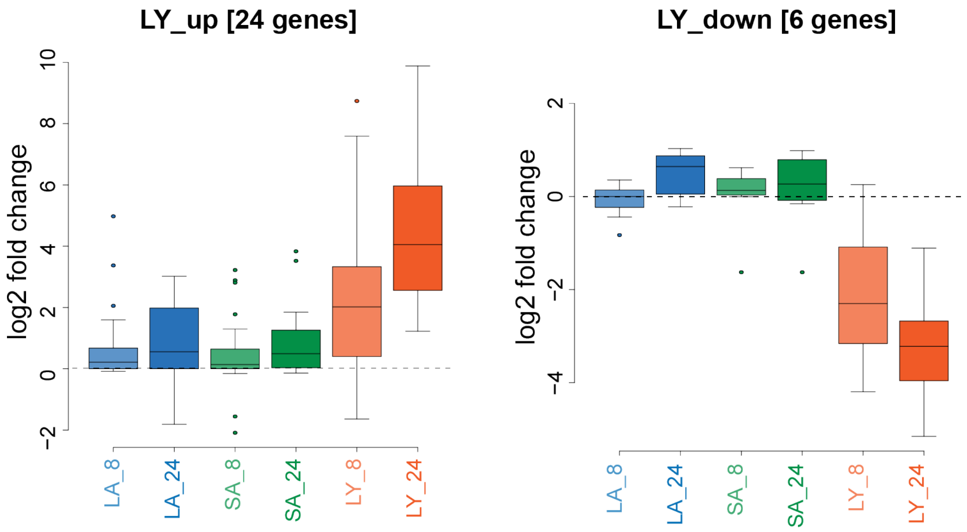

Figure S3. Identification of global and outcome-specific p53 regulatory patterns. *(A)* Number of identified p53 peaks relative to the fraction of peaks containing p53 motifs in indicated KPR tumor types. (*B*) Integration of p53 ChIP-seq and RNA-seq data across KPR tumor types. Numbers represent unique genomic regions bound by p53 and associated with differentially regulated RNAs upon 24h Tam treatment, classified by genomic location of peak, presence or absence of p53, and core or outcome-specific expression pattern. (*C*) p53-restoration-dependent expression changes of 24 upregulated and 6 downregutation genes corresponding to LY-specific p53-bound genomic regions in indicated KPR cells.

Tesfaye et al., Suplementary Figure 4

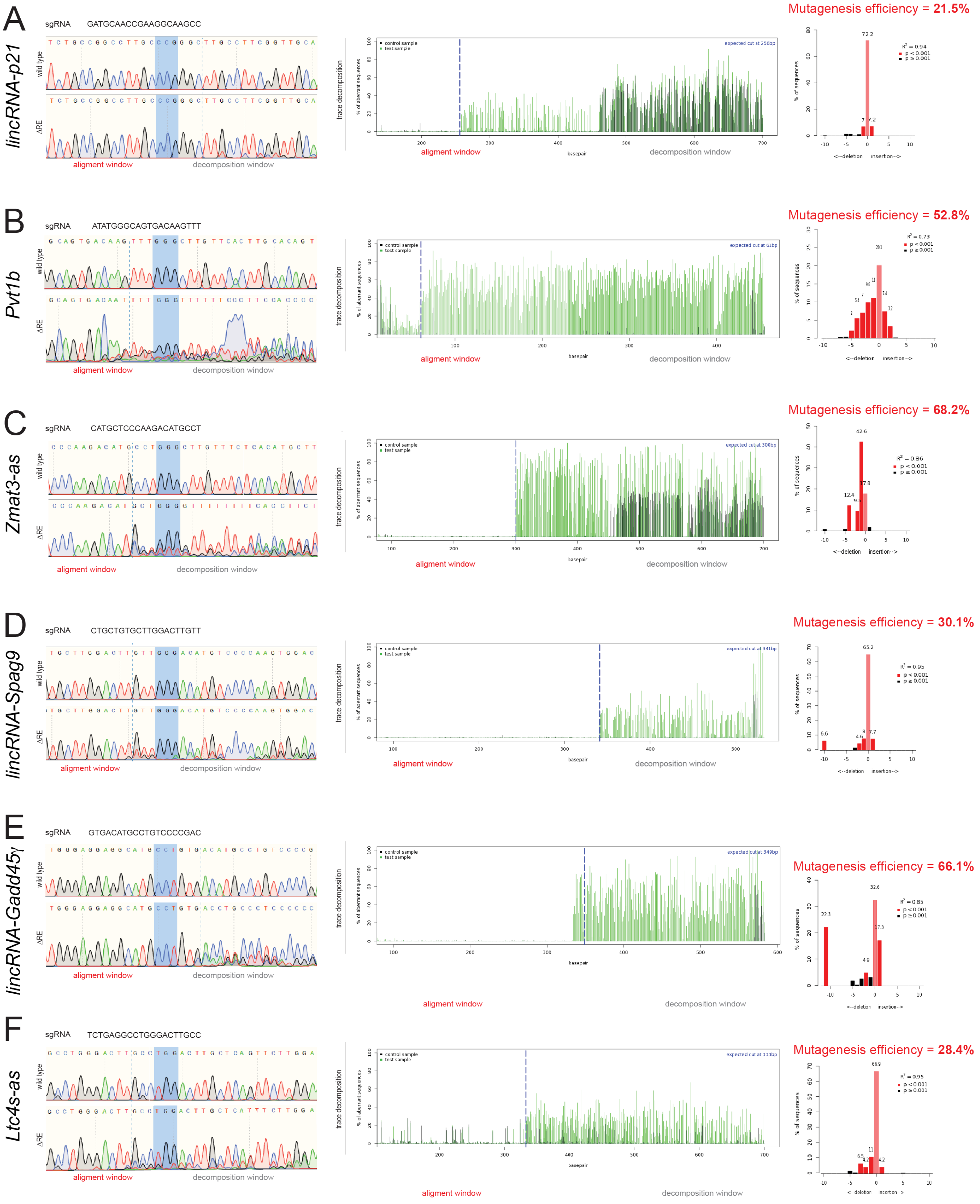

Figure S4. TIDE analysis of p53RE mutagenesis. *(A-F)* TIDE (Tracking Indels by Decomposition) analysis of mutagenesis efficiency of p53REs associated with indicated lncRNAs (*left*) by Sanger sequencing of PCR amplicons from populations of LA1 cells expressing indicated ∆RE gRNAs**.**

Tesfaye et al., Supplementary Figure 5

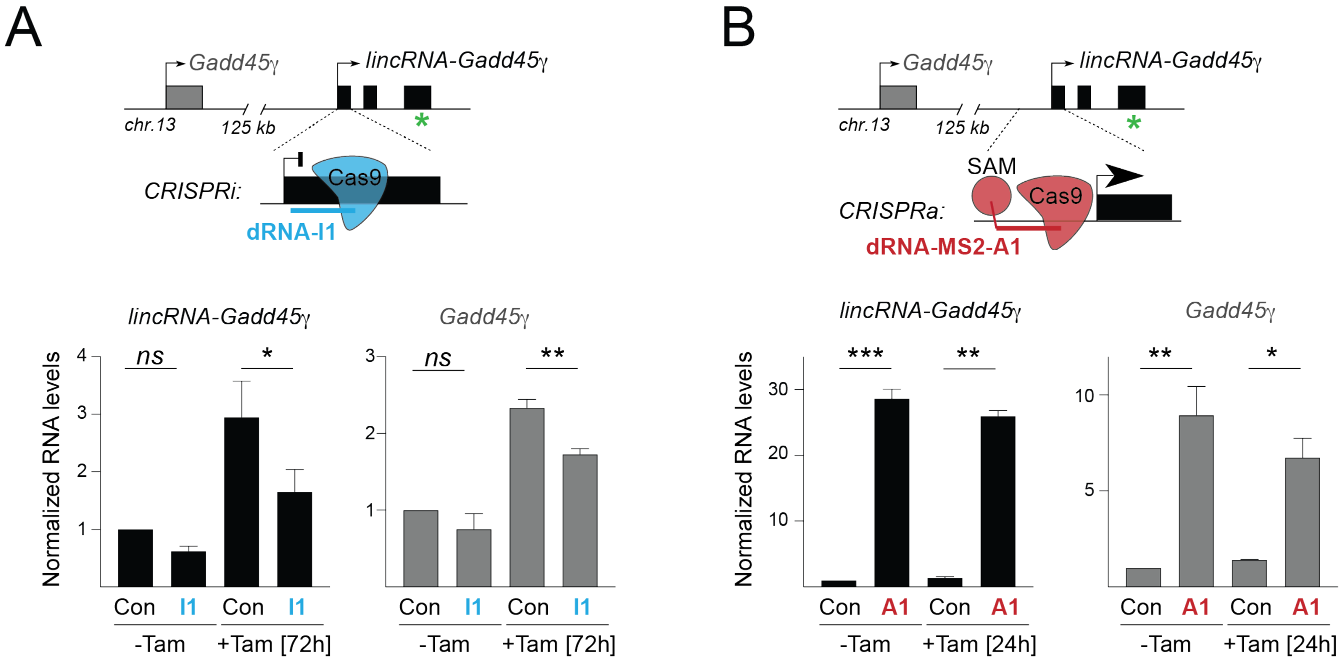

Figure S5. *LincRNA-Gadd45γ* reinforces *Gadd45γ* expression in *cis* during the p53 response to oncogenic stress. *(A) Top*, Design of *lincRNA-Gadd45γ* CRISPR inhibition (CRISPRi) experiment. Bottom, qRT-PCR analysis of *lincRNA-Gadd45γ* and *Gadd45γ* in indicated cells and treatments. Data represent mean ± SEM (n=3 biological replicates). *(B) Top*, Design of *lincRNA-Gadd45γ* CRISPR activation (CRISPRa) experiment. Bottom, qRT-PCR analysis of *lincRNA-Gadd45γ* and *Gadd45γ* in indicated cells and treatments. Data represent mean ± SEM (n=3 biological replicates).

Tesfaye et al., Supplementary Figure 6

A

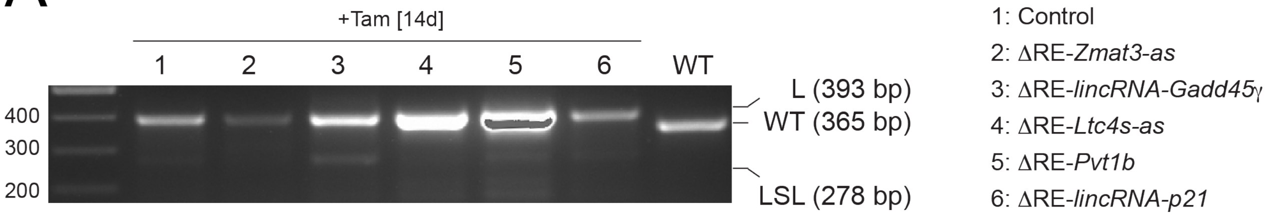

B

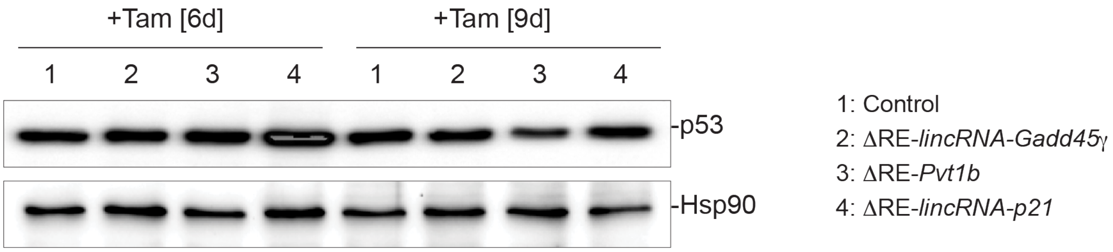

Figure S6. Efficiency of p53 restoration. *(A)* PCR genotyping of p53 alleles (L, recombined, p53 “ON”; WT, wild-type; LSL, unrecombined, p53 “OFF”) in genomic DNA isolated from indicated LA1 cells at 14 days Tam treatment. Data confirm efficient p53 restoration across indicated LA1 cells. Genomic DNA from wild-type (WT) B6 mouse cells as a control. 100 bp ladder for reference. *(B)* Immunoblot showing equal p53 levels across indicated LA1 cells at indicated time-points of Tam treatment. Hsp90 serves as a loading control.
